# Cryochemical synthesis of ultrasmall, highly crystalline, nanostructured metal oxides and salts

**DOI:** 10.3762/bjnano.9.166

**Published:** 2018-06-12

**Authors:** Elena A Trusova, Nikolai S Trutnev

**Affiliations:** 1 Institution of Russian Academy of Sciences, A.A. Baikov Institute of Metallurgy and Materials Science, 49 Leninsky Pr., Moscow 119334, Russian Federation; 2 Moscow Polytechnic University, 38 Bolshaya Semenovskaya Str., Moscow 107023, Russian Federation

**Keywords:** cryochemical synthesis, cryosols, nanocrystalline metal oxides, nanostructured powders

## Abstract

In the present investigation, the cryochemical approach was used for the improved synthesis of nanocrystalline metal oxides (e.g., NiO, Fe_2_O_3_, CeO_2_) and NaNO_3_ salt. It was shown that the solutions and sols can be treated with a liquid nitrogen stream (−196 °C) to increase the powder dispersity by 3–18 times and to increase their specific surface area by an order of magnitude. The proposed approach also reduces the agglomeration of the nanoparticles, and at the same time, results in NiO, Fe_2_O_3_ and CeO_2_ crystallite sizes of less than 10 nm (quantum dot size regime). The diameter of NaNO_3_ salt crystallites could also be reduced to ≤50 nm by freezing in a liquid nitrogen atmosphere, which is a significant improvement over analogous salts obtained by traditional methods (average diameter 300–1000 nm). The characterization of the obtained nanopowders was carried out using X-ray diffraction, transmission electron microscopy, surface area measurements and diffusion aerosol spectrometry (DAS). It was determined that the addition of 3–15 wt % of NaF to the NaNO_3_ solution prior to its cryogenic treatment results in a further decrease in the particle size of the obtained crystalline salt. NaF creates a protective coating with a thickness of 2–3 nm on the surface of NaNO_3_ crystallites, preventing their association. The results obtained show that the cryochemical processing of the solutions during the preparation phase of production allows nanopowders to be obtained with improved morphological and textural characteristics without significant increase in technical development costs.

## Introduction

In recent decades, nanostructured raw products have become particularly in demand for obtaining many of the new functional and structural materials. In a 1962 publication, theoretical justification was given for the use of low (negative) temperatures for studying the mechanisms behind chemical processes and the peculiarities of reaction kinetics in the low-temperature region in order to improve the synthesis of ultra-clean materials [1]. A cryochemical approach to study the spontaneous chemical interaction of a high-temperature substance with a gas or liquid at liquid nitrogen temperature (−196 °C) or lower appeared in the second half of the 20th century [2–4].

The increased use of negative temperatures (4–100 K) has led to the further development of new materials for use in nanochemistry and nanotechnology. Such materials contribute to the development of new types of small-sized electronic devices, optical materials with improved properties, fine-grained ceramics, and they promote progress in the chemical industry and have applications in medical and biological research. Sergeev et al. have shown that the particle size is an active variable that together with other thermodynamic variables determines the state and reactivity of the system, whereby nanoparticles can promote chemical reactions which are not possible for compact states [5–7].

Most often, aqueous solutions are used; however, solutions in organic solvents (e.g., acetic acid, benzene, *tert*-butanol, and toluene) can also be used when reasonable equilibrium pressure is applied in the solid state at low temperature. The low solubility of many inorganic salts in these solvents limits their application in cryochemical processing. Along with aqueous solutions, in the last few years the freezing of colloids (gels, suspensions) and precipitates has been increasingly used [8].

As a rule, salts solutions are treated by fast cooling [9]. For example, aqueous solutions of Al- and Fe-sulfates were used to obtain highly dispersed Al_2_O_3_ and Fe_2_O_3_ crystallites by a freeze-drying technique. The formation and growth of chain-like aggregates of crystallites was shown as a process followed the surface diffusion mechanism. It was observed that the orientation of the chain aggregates was related to the ice structure formed during freezing [10].

The cryoprocessing of liquid media, solutions and colloids can be used to limit the growth and agglomeration of the formed particles, to stabilize their surface, and thus, to obtain discrete particles of predetermined size that are several nanometers in diameter in the form of clusters or crystallites with an almost ideal lattice. It has been shown that when a colloid undergoes nitrogen cryotreatment, the formed nanoparticles, having a high surface energy, are characterized by a low degree of aggregation due to the fast-moving formation of the particle surface [11].

The high activity of nanostructures in solid-phase processes is a valuable property in ceramics sintering from them. The use of cryotechnological methods leads to an improvement in the structural properties of raw materials, which is very important for creating fine-grained ceramics with specified structure and properties. Fine-grained ceramics need a homogenous structure to provide its unique properties of plasticity, high strength, wear-resistance, etc. [12].

We previously reported on the synthesis of nanostructured catalysts consisting of NiO nanocrystals (4.1–4.7 nm) incorporated into the pore space of mesoporous titanium silicate [13].

We developed a method for the preparation of ultradispersed NiO based on the combination of the microemulsion method and cryochemical technology. NiO crystallites were obtained by cryotreatment of the metal-containing colloid and were incorporated in the titanium silicate (Ti_0.03_Si_0.97_O_2_) pores. It was found that chemical bonds are formed between the silicate surface and NiO crystallites, which leads to new physicochemical properties of the material. The high activity of NiO nanoparticles was indicated in the formation of a 2D interface layer which included Ni–O–Si bonds on the surface of the mesoporous silicate. It was found that the composite system prepared by cryotreatment of the emulsion showed a higher hydro-desulfurization catalytic activity by several orders of magnitude as compared to that of the same composition prepared directly from an as-prepared microemulsion.

In [14] we reported on a method for producing metal oxides (CeO_2_, Fe_2_O_3_ and NiO) with a crystallite size less than ≤10 nm by a combination of the sol–gel method and cryotechnology. The essence of the method is to create soft conditions for the formation of Ce-, Fe- and Ni-oxide nanocrystallites, and its special feature is the use of cryogenic processing (liquid N_2_, −196 °C ) of metal-containing water–organic sols. In addition, we have patented a device for the production of cryochemical crystalline substances from solutions and suspensions using a swirler [15].

In this paper, we investigated the possibility of obtaining metal oxide and salt nanopowders from microemulsions and solutions using a cryochemical approach. It was shown that treatment of the stock solutions and sols with a liquid nitrogen stream (−196 °C) can lead to an increase in powder dispersity by many times. The average crystallite size of all synthesized powders was less than 10 nm. Cryochemical processing of the solutions during the production allows nanopowders to be obtained with improved morphological and textural characteristics without significant increase in technological cost.

## Experimental

### Preparation

The process of obtaining nanopowders via cryotreatment consists of several basic steps. First, the salt solutions were prepared starting with a 30% salt solution in deionized water to produce sodium nitrate nanopowders. The combined method, including a modified sol–gel synthesis of the colloids and their cryogenic processing, was used in the synthesis of nickel, iron and cerium oxide nanopowders. In this case, cerium, iron and nickel nitrates were used as metal sources for the synthesis of metal-containing sols. The sol synthesis was carried out at 60–80 °C using a magnetic stirrer (500–600 rpm). *N*,*N*-dimethyloctylamine (DMOA), monoethanolamine (MEA) or hexamethylenetetramine (HMTA) were used to obtain and stabilize the sols.

The resulting liquids were fed into a cryogranulator where they were sprayed into a container with liquid nitrogen (−196 °C) using a special jet injector with a cylindrical nozzle.

The spray torch was sent to the liquid nitrogen container, where the formation of cryogranules occurred at a high cooling rate. The instantaneous freezing of the droplets produced by spraying led to the formation and crystallization of solid microgranules. The resulting cryogel was vacuum-freeze-dried to remove the liquid phase at a temperature of 80–100 °C and a pressure of ≤3 × 10^−2^ mmHg. The heat treatment of the as-prepared product was continued in an oven in air at a temperature of 500 °C for 1–3 h, and as a result, well-dispersed NiO, Fe_2_O_3_, CeO_2_ and NaNO_3_ nanopowders were obtained.

**Figure 1:**
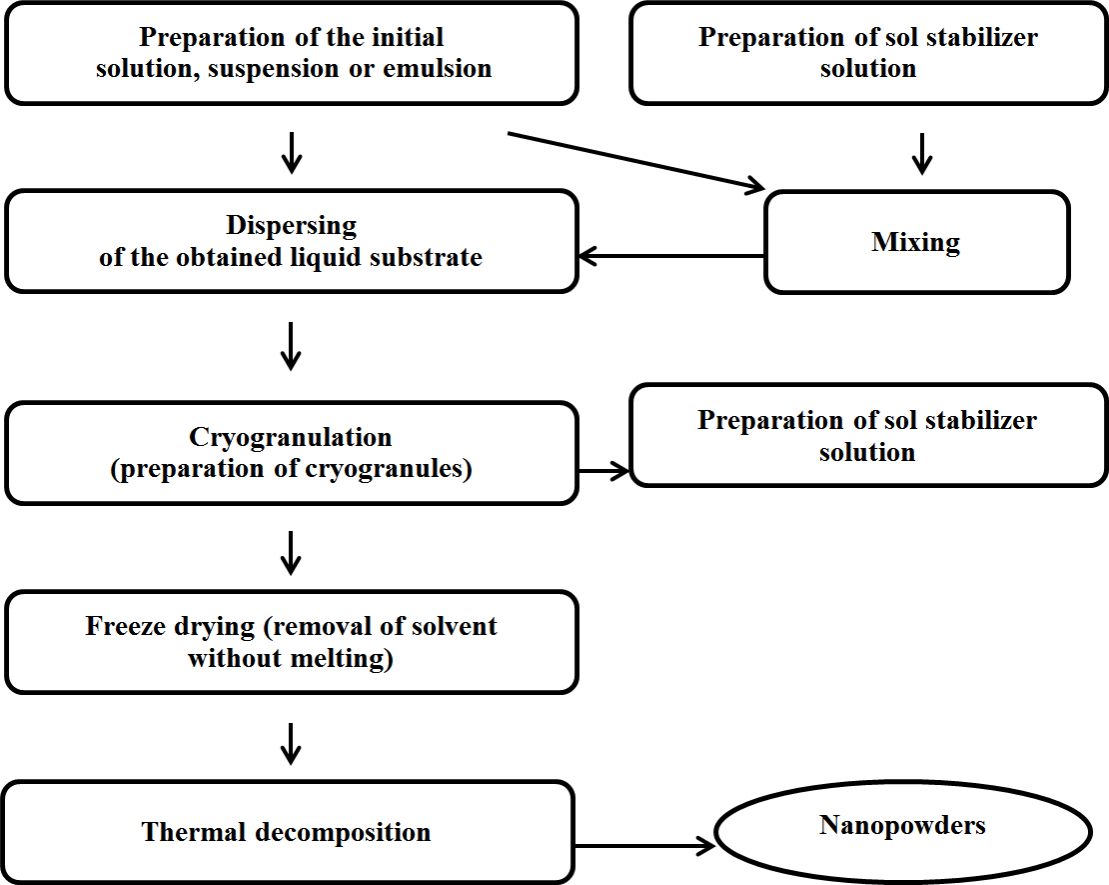
Steps of the cryochemical synthesis of nanopowders.

Figure 1 provides a description of the synthesis process of nanopowders using cryotreatment of solutions or colloids. The salt solutions, sols and microemulsions were used as liquids for further cryogenic processing. In the synthesis of metal oxide nanopowders, the starting salt solutions were mixed with a solution of DMOA in acetylacetone, whereby a stock solution was obtained. To obtain the sodium nitrate nanopowder, the salt solution was sent to the dispersion immediately after production. The resulting solutions or colloids were sprayed using a hydraulic jet injector with a swirler, directing the spray torch into the liquid nitrogen environment (−196 °C), where cryogranulation took place. In the next step, the granules were exposed to freeze-drying and calcination, resulting in the production of metal oxide nanopowders (Ce, Fe or Ni), as well as highly dispersed sodium nitrate powder.

### Characterization

The phase composition and morphology of the nanopowders obtained were investigated by X-ray diffraction methods (XRD, DRON-3M (Russia) and XRD-6000 Shimadzu (Japan)), transmission electron microscopy (TEM, LEO 912 AB Omega Carl Zeiss and Philips EM-301) and diffusion aerosol spectrometry (DAS, model 2702, Aeronanotech). The specific surface area was determined using the BET method on a NOVA 2200 instrument. Elemental analysis for the presence of residual carbon and nitrogen was carried out using an inductively coupled plasma atomic emission spectrometer (ICP-MS, Optima-5300). It was shown that the content of residual carbon and nitrogen in the powders did not exceed 0.08 and 0.07 wt %, respectively. The NiO nanopowder was characterized by X-ray photoelectron spectroscopy (XPS, PHI5500 Versa Probe II) with a monochromatic Al Kα X-ray source (*h*ν = 1486.6 eV, 50 W).

## Results and Discussion

**Figure 2:**
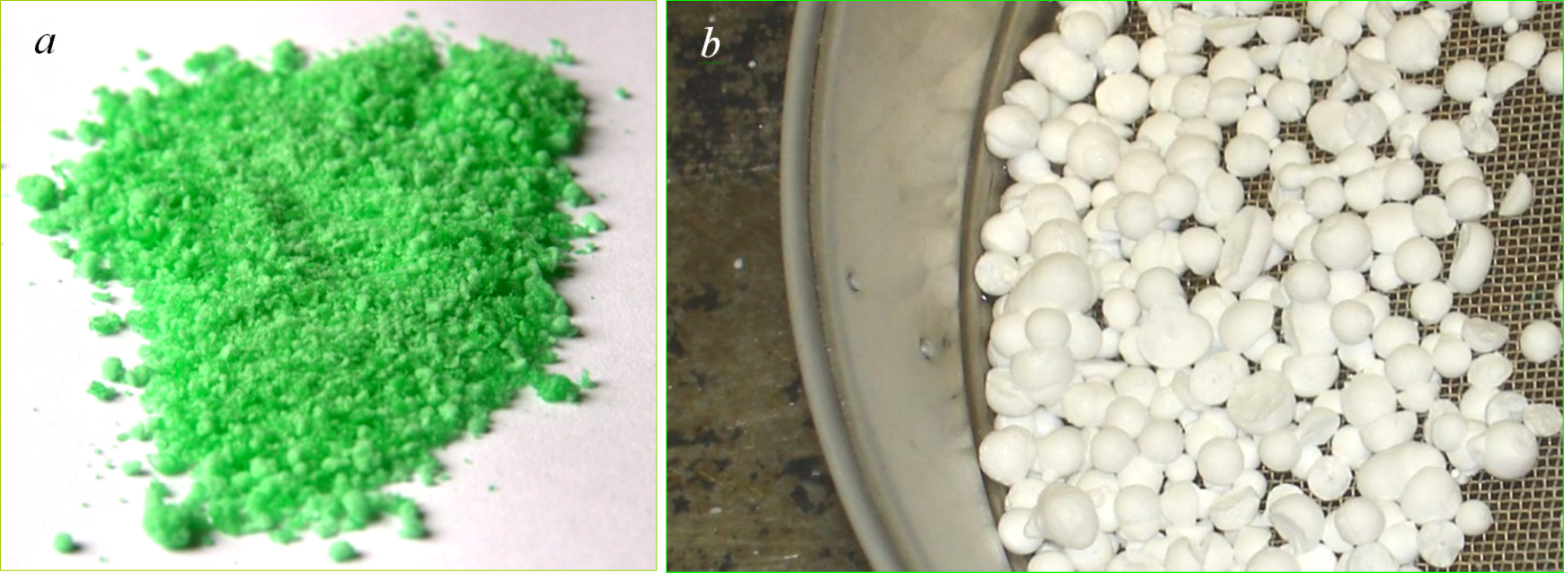
Photographs of the cryogel, which is a precursor of the NiO nanopowder (a) and the cryogranulate, which is a precursor of the sodium nitrate powder (b).

Figure 2 shows photographs of a Ni-containing cryoaerogel consisting of particles with diameter of 0.1–1.5 mm (Figure 2a) and a cryogranulate material, which is a precursor of sodium nitrate powder (Figure 2b).

**Figure 3:**
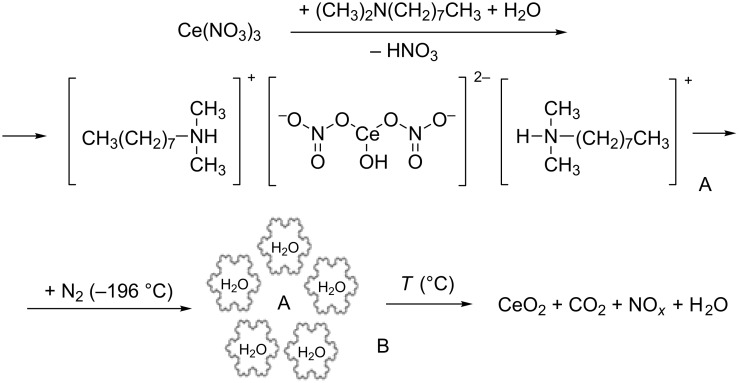
Scheme of CeO_2_ nanoparticle formation, including the cryotreatment step (liquid N_2_, −196 °C ) on the as-prepared sol. A is the sol intermediate formed from the interaction of hydrolyzed Ce^3+^ ions with DMOA. B is the cryogel formed from the interaction of liquid nitrogen with A.

The mechanism of metal oxide nanoparticle formation under of sol–gel synthesis in combination with cryotreatment of the sol is presented in Figure 3 using the example of the ceria nanopowder. The process under consideration involves the interaction of hydrolyzed Ce^3+^ ions with DMOA to form the sol intermediate, A. Then, the cryogel (labeled as B in Figure 3) is formed during the treatment of sol A with liquid N_2_ (−196 °C ). The resulting water crystallite shell prevents the agglomeration of the sol particles.

**Scheme 1:**
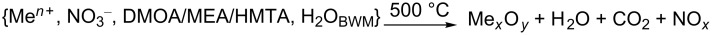
Scheme describing the formation of crystalline metal oxides with water, carbon dioxide, and NO*_x_* as byproducts. Me*^n^*^+^ – metal ions; NO_3_^−^ – metal source, ligand; BWM – bound water molecules.

The obtained organic–inorganic cryogel powders consisted of capillary porous granules (Figure 2) and contained the stock solution components in their structure, i.e., Ni^2+^, Fe^3+^ or Ce^3+^ ions, as well as sol stabilizers and bound water molecules (Scheme 1). During the subsequent heat treatment in air, the following processes took place: dehydration of the hydrolyzed components, desorption of organic components of the cryogel, and decomposition of the organic–inorganic composite oligomer complex (360–380 °C ) to form crystalline metal oxides as a product in addition to water, carbon dioxide, and NO*_x_* (Scheme 1) as a result of ligand oxidation.

The synthesized cryogels were treated with heat for 1 h at 500 °C. The diameter of the resulting metal oxide powders was many times smaller (3–18) than for materials prepared without cryotreatment of the sols (Table 1). Apparently, the beginning of crystallization and the formation of the nanoparticle surface occurred even under the ice shell in the cryosols.

**Table 1:** The average diameter (*D*) of the crystallites of the obtained powders (X-ray diffraction data).

Powders	*D* (nm)	
	from sols prepared with cryotreatment (this work)	from sols prepared without cryotreatment (from [14])

NiO	4	≥70
Fe_2_O_3_	8	≥30
СеО_2_	7	≥20
NaNO_3_	50	≥500

**Figure 4:**
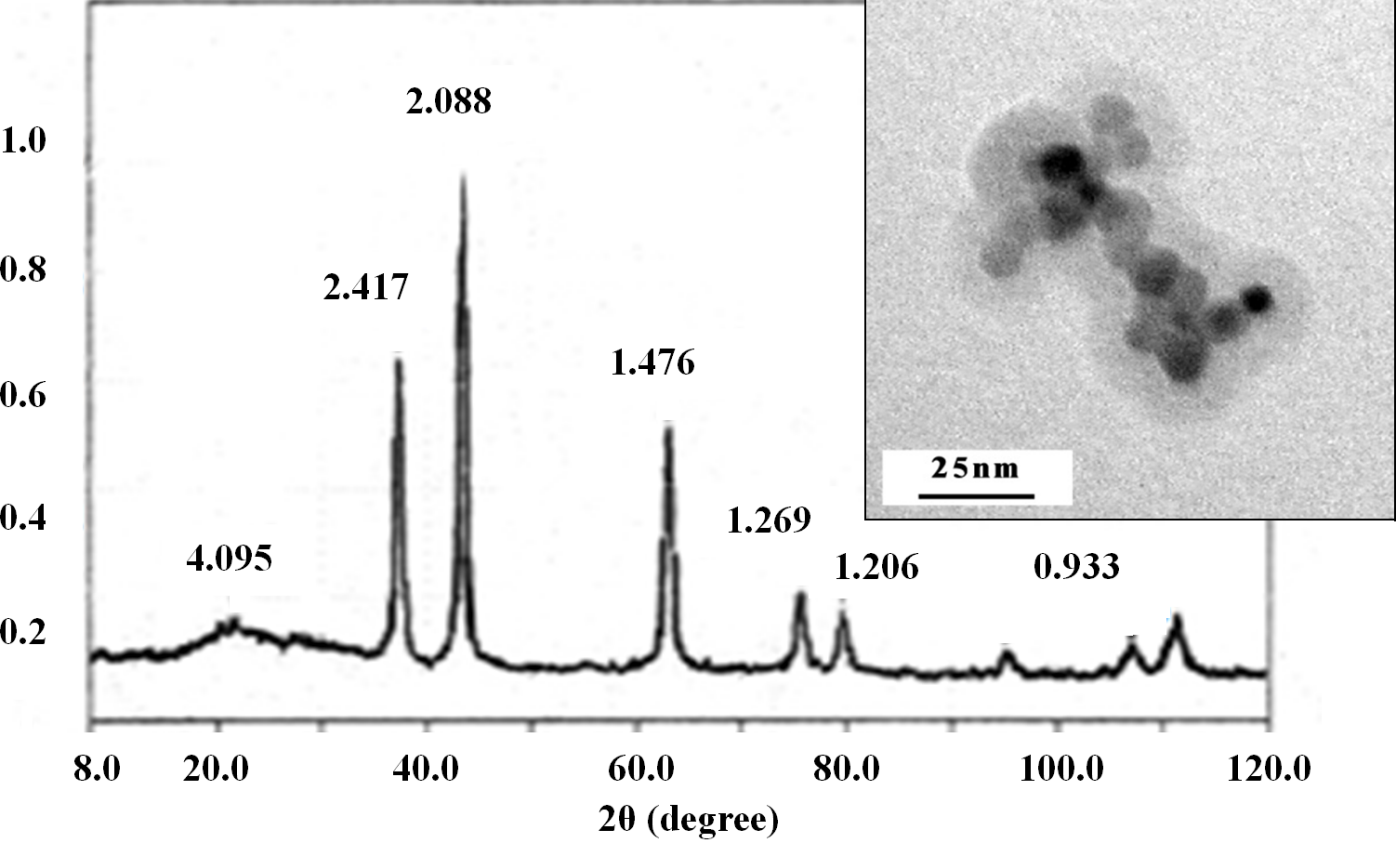
XRD pattern (DRON-3M, Russia) and TEM image (insert) of the NiO nanopowder.

**Figure 5:**
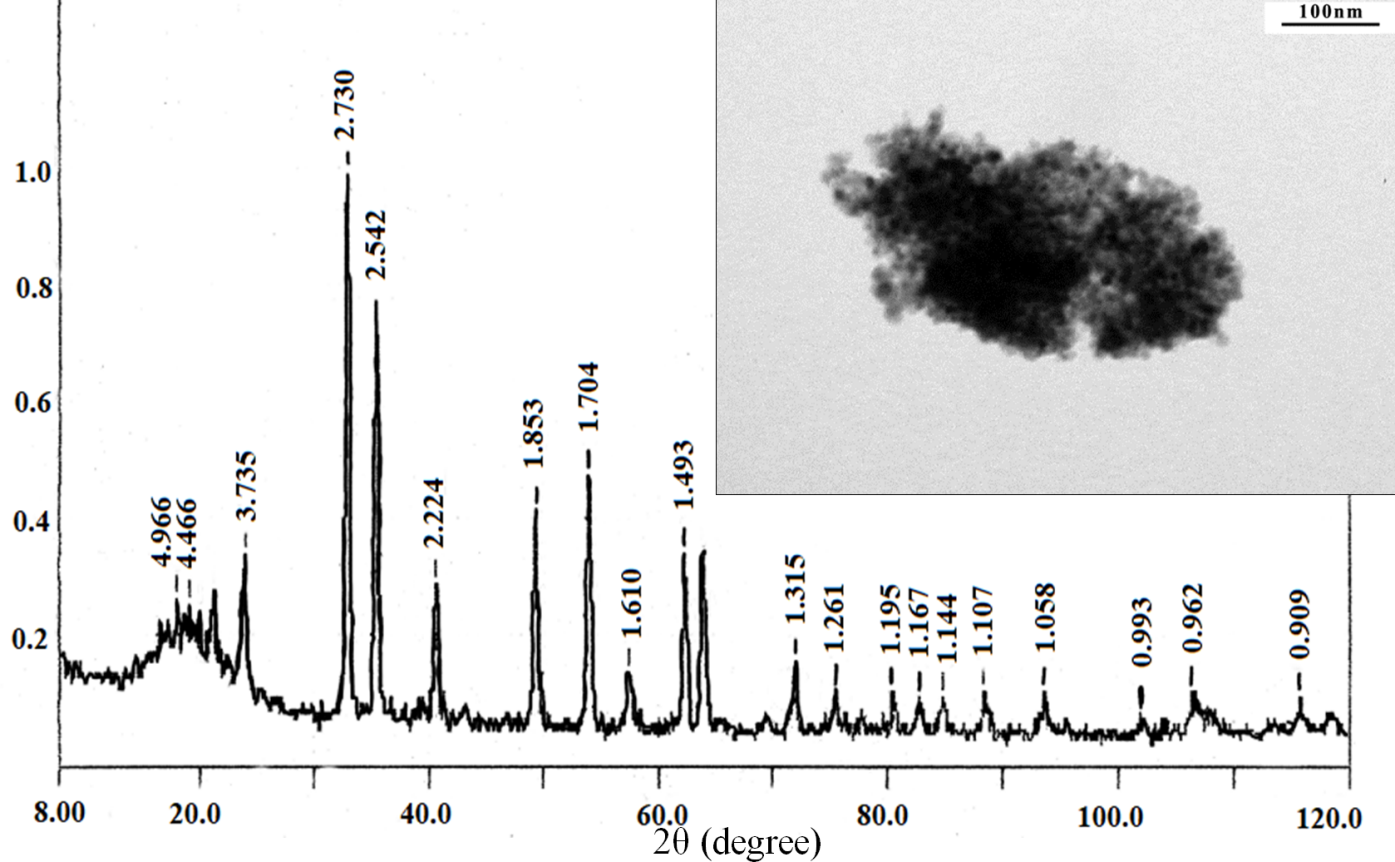
XRD pattern (DRON-3M, Russia) and TEM image (insert) of the Fe_2_O_3_ (hematite) nanopowder.

According to the XRD data, all metal oxide powders were nanodispersed well-crystallized systems, with microdeformations of the lattice as a rule not exceeding 0.2 (0.04–0.05)%. The XRD patterns show that the ultradisperse powders of NiO (JCPDS Card No. 47-1049) and Fe_2_O_3_ (hematite, JCPDS Card No. 88-2359) obtained from cryoaerogels were crystallized to 100% (Figure 4 and Figure 5). The average crystallite size was calculated by the Rietveld method to be 4.1–4.7 and 8.0–8.6 nm, respectively (Table 1).

According to the TEM data for the NiO powder (Figure 4, insert) most of the particles have a diameter of 4–5 nm and are of spherical or hexahedral shape. The results of the TEM study of the Fe_2_O_3_ (hematite) nanopowder also correspond well to the calculated data from the XRD results; the crystallite size did not exceed 10 nm (Figure 5, insert).

**Figure 6:**
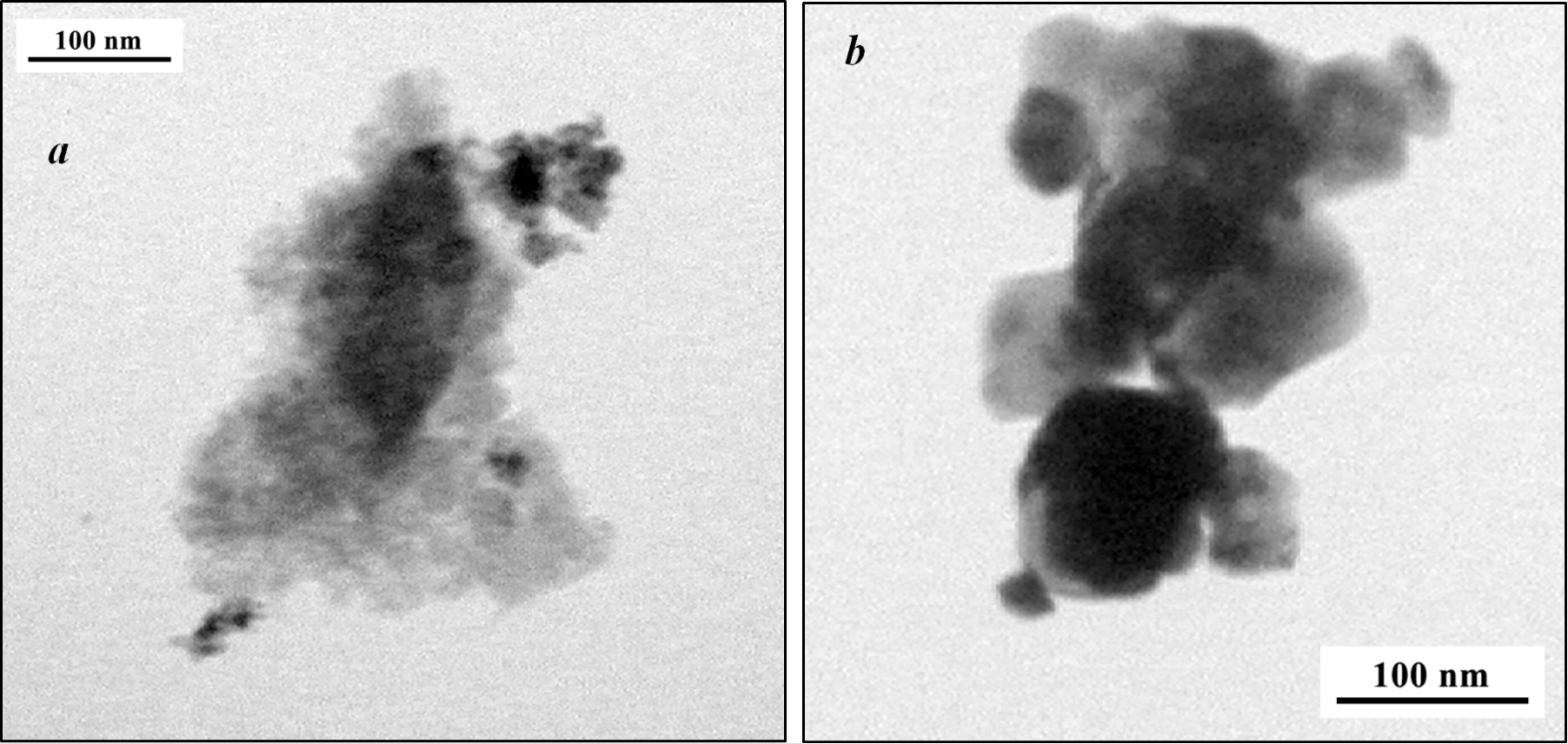
TEM images of CeO_2_ nanopowder obtained from the cryosol (a) and CeO_2_ nanopowder obtained from a freshly prepared sol without cryotreatment (b), showing that the cryotreatment results in a better-dispersed nanopowder with smaller crystallite size.

For the ceria sample, it was shown that the cryotreatment of the sols led to an increase in the BET surface area of the nanopowder by more than an order of magnitude. The BET surface area of the nanopowder obtained from the cryosol was 140 m^2^/g, while using a freshly prepared sol without cryotreatment had a BET surface area of 10 m^2^/g. The TEM data show that the CeO_2_ nanopowder obtained from the cryosol consisted of crystallites with dimensions less than 10 nm (Figure 6a). The CeO_2_ nanopowder obtained from the as-prepared sol consisted of crystallites with a wide size distribution, from 20 to 90 nm (Figure 6b).

**Figure 7:**
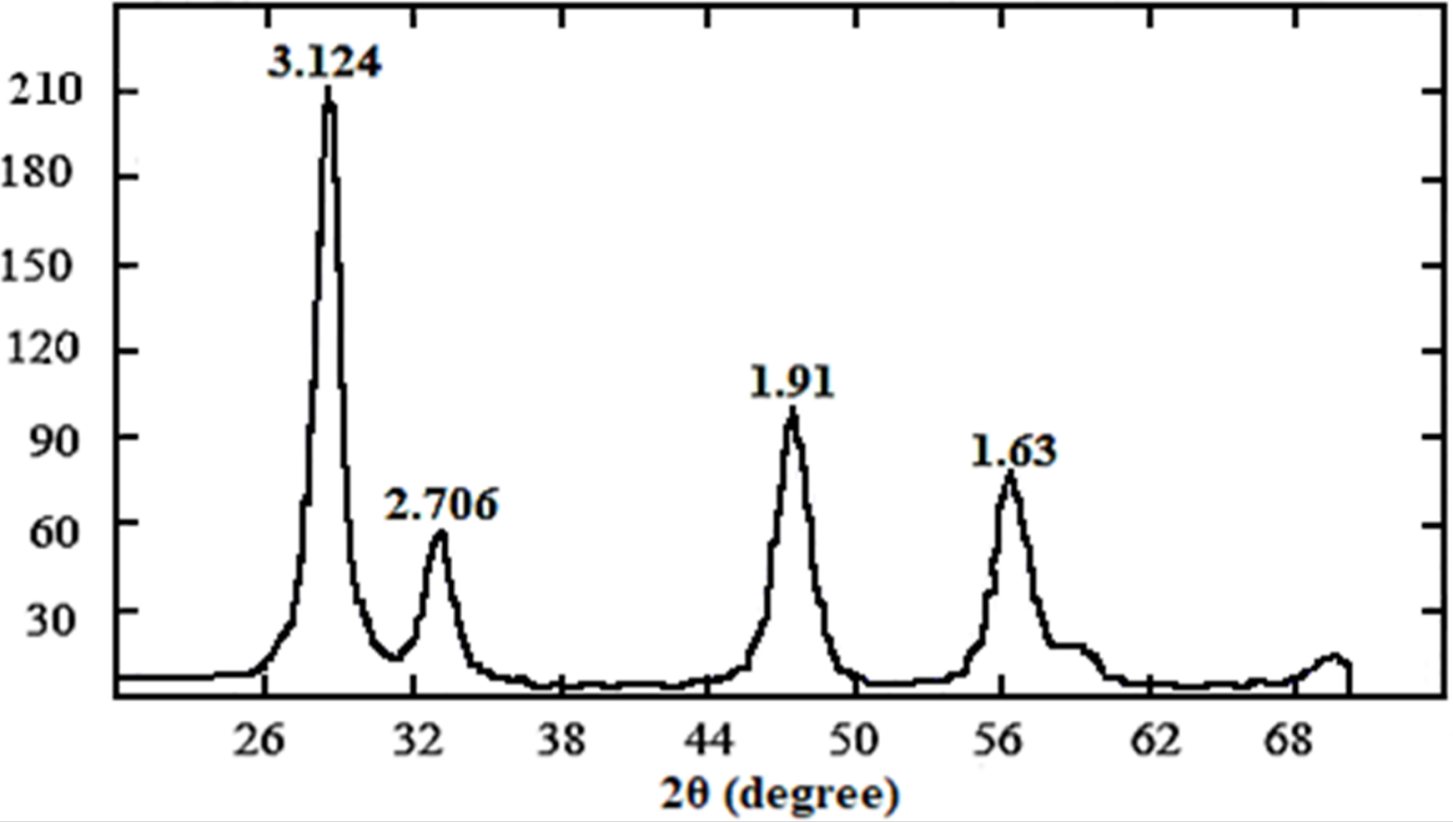
X-ray diffraction pattern for a CeO_2_ (JCPDS Card No. 43-1002) nanopowder obtained from a cryosol (XRD-6000 Shimadzu, Japan).

The XRD pattern for the CeO_2_ powder obtained from a cryosol (Figure 7) shows an highly dispersed crystalline state, which was confirmed by the TEM data (Figure 6a). The broadened reflexes confirm the high dispersity of the CeO_2_ crystallites.

**Figure 8:**
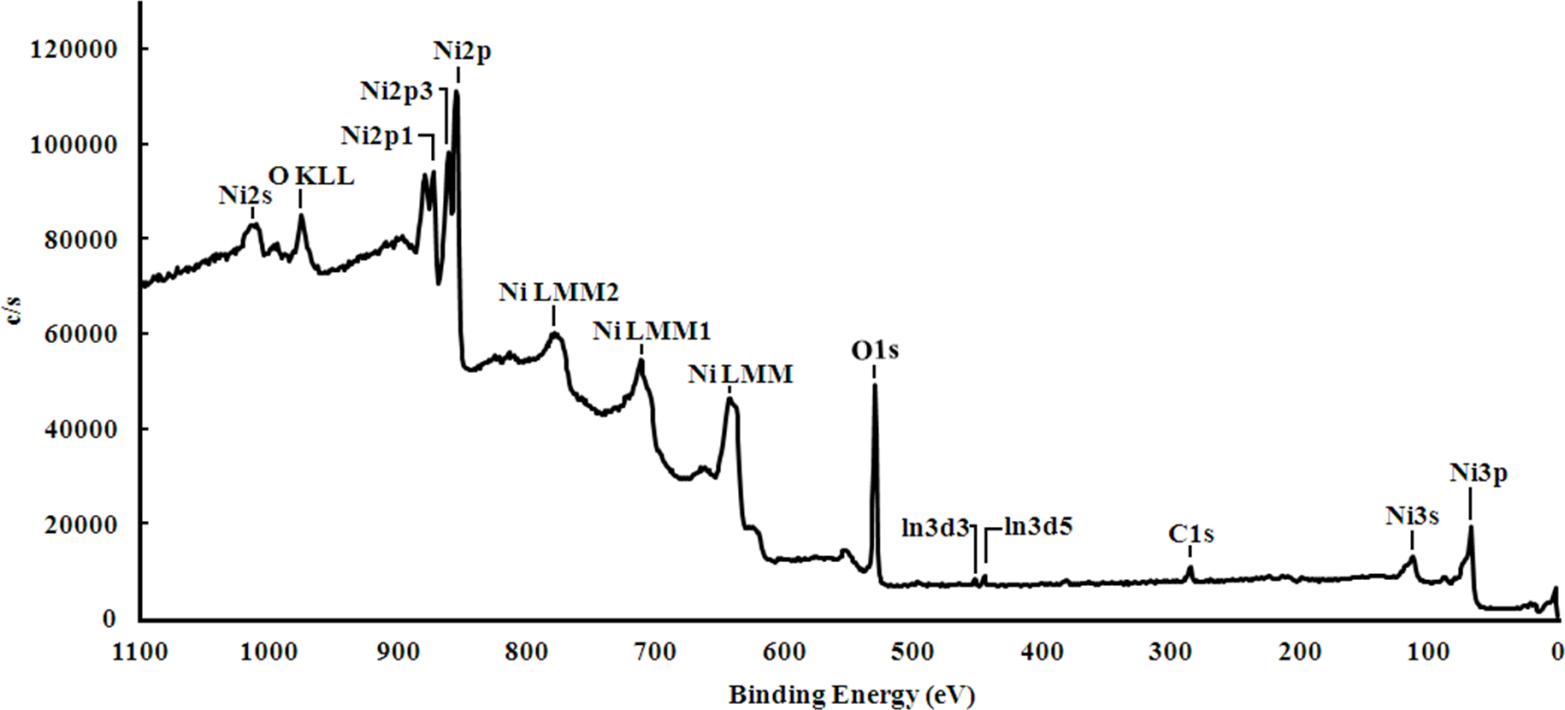
Collected XPS spectrum of NiO nanopowders synthesized using the cryochemical method.

**Figure 9:**
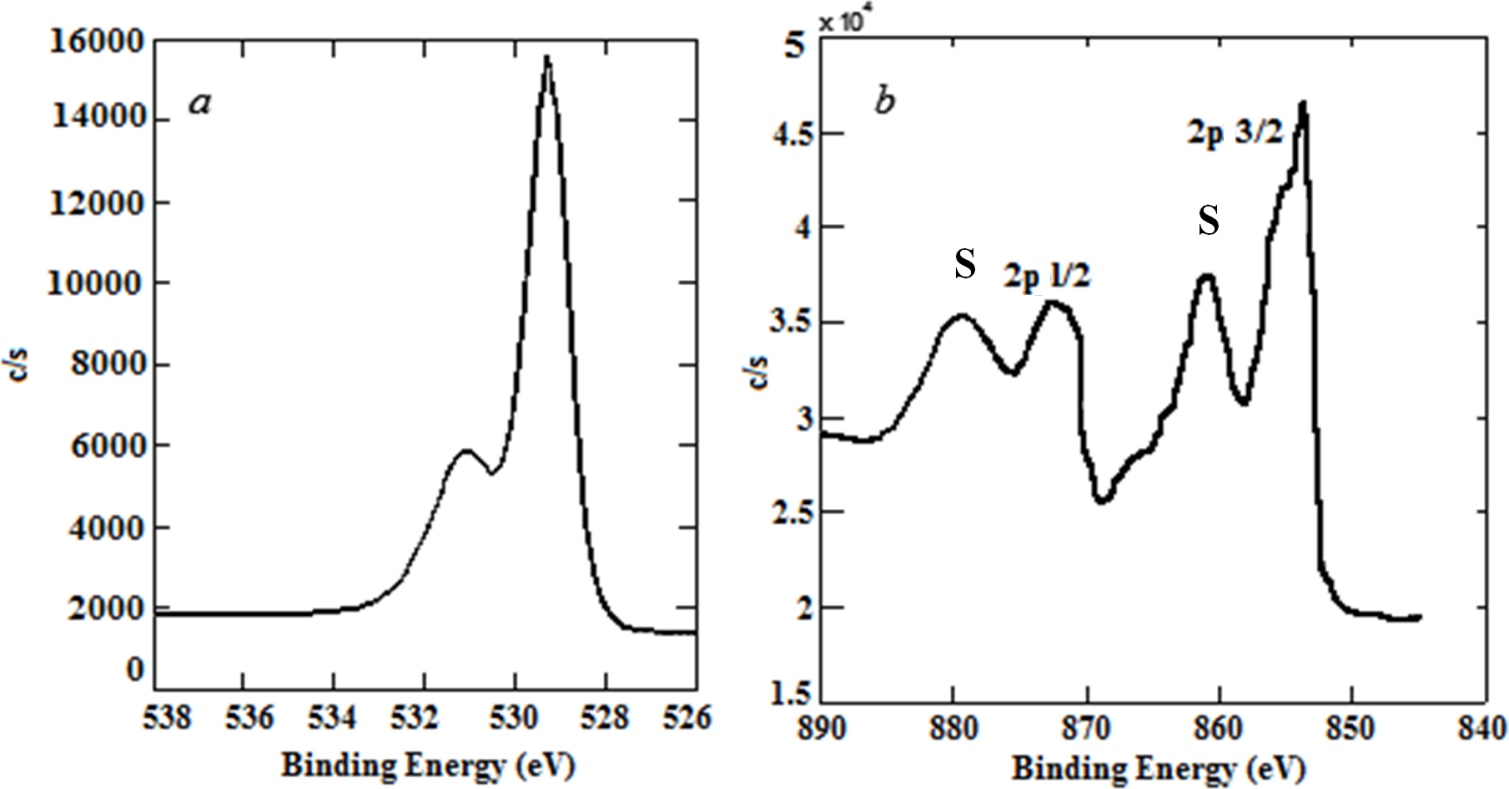
XPS data for NiO nanopowders synthesized using the cryochemical method: main O 1s peak is associated with structural oxygen (a) and the Ni 2p peak, containing its characteristic satellite peaks denoted by the letter S is given in (b).

Figure 8 shows the collected XPS spectrum of the NiO nanopowder obtained by using the cryochemical method. The main O 1s peak from structural oxygen is located at a binding energy (BE) of ≈529.4 eV; whereas the presence of the Ni 2p_3/2_ peak with a BE of ≈853.8 eV clearly indicates NiO (Figure 9a). The shape of the Ni 2p peak with characteristic satellite peaks (denoted by the letter S) also corresponds to NiO (Figure 9b) [16].

**Figure 10:**
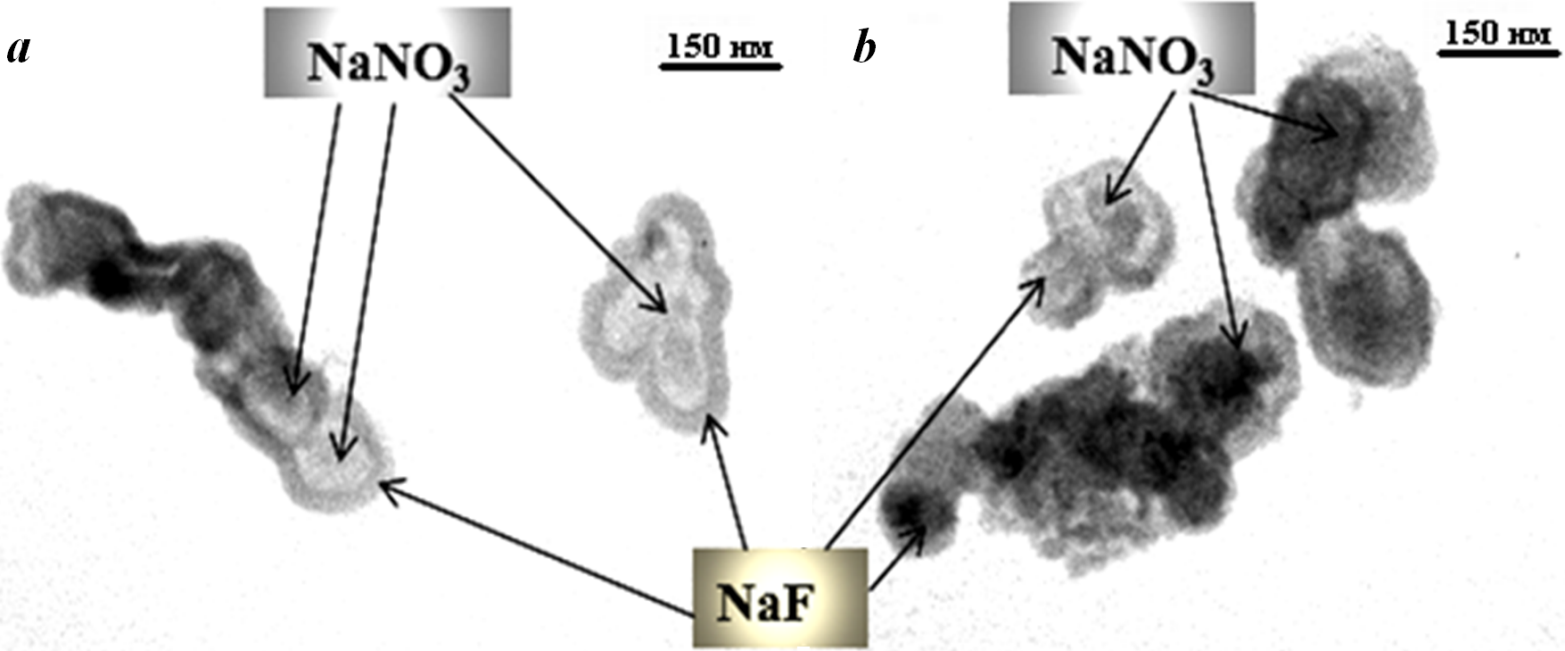
TEM images of sub-micrometer-sized NaNO_3_–NaF powders containing 3 wt % (а) and 15 wt % (b) of NaF additive. The scale bar represents 150 nm.

The problem with the production of sub-micrometer-sized nitrate powders resistant to caking was also solved through the use of cryochemical process and the addition of NaF in an amount of 3 or 15 wt %. According to the TEM data (Figure 10a,b), the difference in NaF content in the powder practically does not affect the particle size, which was 100–200 nm. The micrographs clearly show that practically every nitrate crystallite is coated with a coating of 2–3 nm thickness.

**Figure 11:**
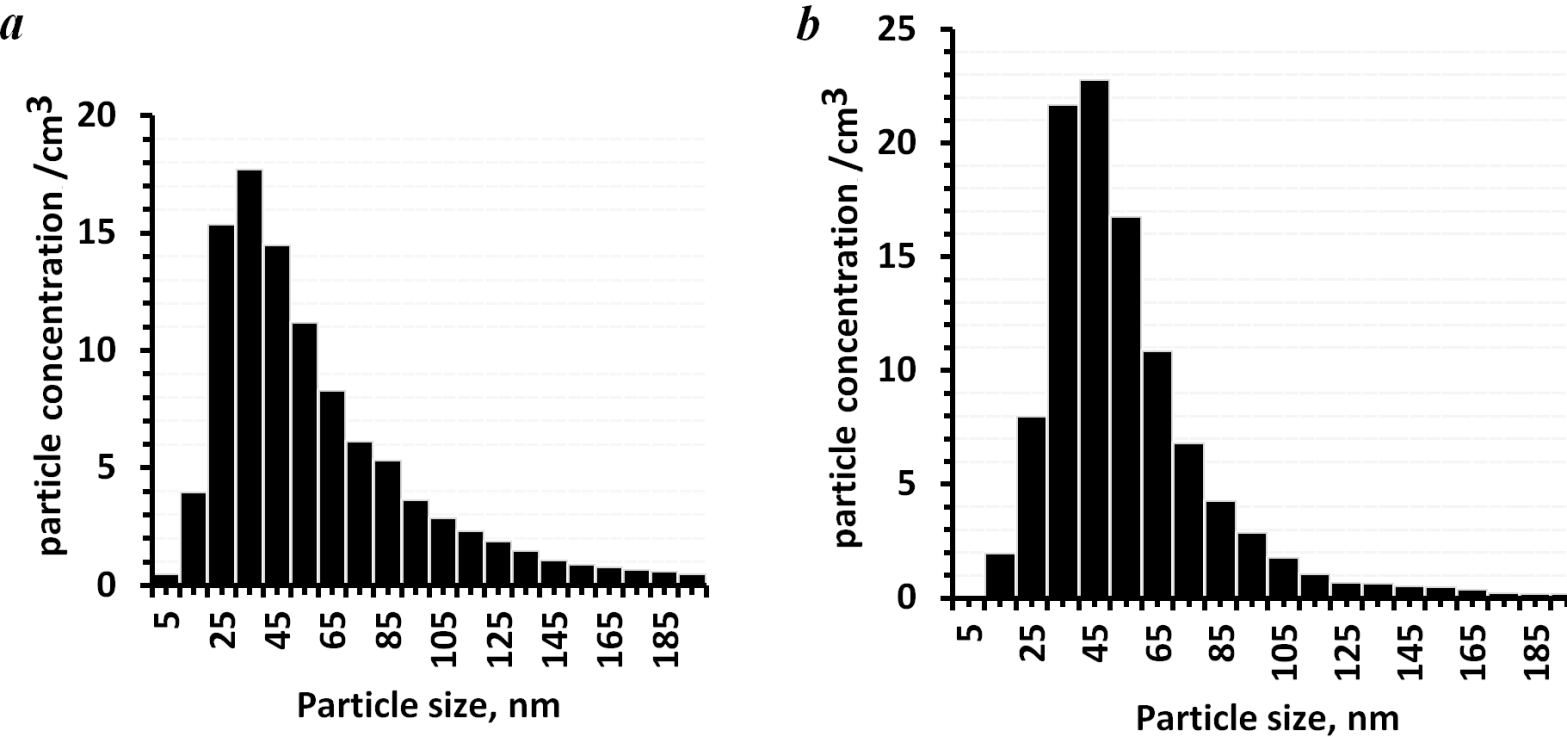
Histograms of the particle size distribution for NaNO_3_ powders synthesized with the addition of 3 wt % (а) and 15 (b) wt % NaF.

Using the DAS method, it was shown that a powder containing 15 wt % of NaF had a narrower particle size distribution (Figure 11a,b). For the powder containing 3 wt % of NaF, 72% of the particles had a diameter of 15–85 nm. When the powder contained 15 wt % of NaF, then 72% of the particles had a diameter of 25–70 nm. Apparently, the F**^−^** ions in the sodium nitrate solution (NaNO_3_) prevent both the growth of crystallites and their agglomeration.

## Conclusion

In this work it was shown that inclusion of a cryotreatment step performed on the stock solutions of salts and sols in a technological synthesis scheme allows nano- and sub-micrometer-sized metal oxide powders and salts with improved morphological and textural characteristics to be obtained without significantly increasing the cost of the technology. Namely, the treatment of stock solutions and sols with a liquid nitrogen stream (−196 °C) was shown to increase the dispersity of the powders by 3–18 times. The average crystallite size of all synthesized metal oxide powders was less than 10 nm. The BET surface area of metal oxide nanopowders synthesized via cryochemistry techniques was shown to increase by more than an order of magnitude. When the cryoeffect is combined with NaF additives, it is possible to obtain highly dispersed (submicrometer) powders of nitrates. In this case, NaF creates a protective coating with a thickness of 2–3 nm on the surface of NaNO_3_ crystallites, preventing their association. The incorporation of the proposed method into existing technological process will expand the assortment of nanomaterials and other highly dispersed powders of metal oxides and salts available. Such improved nanomaterials could be further incorporated in a wide range of new functional materials with specified properties for the production of fine-grained ceramics and special-purpose materials. On the basis of the developed technique, an experimental technological process has been created with high-throughput productivity of 0.5–0.8 kg/h.

## References

[1] McGee H A, Martin W J (1962). Cryogenics.

[2] Shlyakhtin O A, Tretyakov Y (1997). Mater Technol (Abingdon, U K).

[3] Tretyakov Y D, Oleynikov N N, Shlyaktin O A (1997). Cryochemical technology of advanced materials.

[4] Klabunde K, Sergeev G (2013). Nanochemistry.

[5] Sergeev G B (2003). J Nanopart Res.

[6] Sergeev G B (2006). Cryochemistry of Metal Atoms and Nanoparticles. Nanochemistry.

[7] Sergeev G B, Shabatina T I (2008). Colloids Surf, A.

[8] Shlyakhtin O A, Oleynikov N N, Tretyakov Y D, Lee B, Komarneni S (2005). Cryochemical Synthesis of Materials. Chemical Processing of Ceramics.

[9] Xu R, Xu Y (2017). Modern Inorganic Synthetic Chemistry.

[10] Johnson D W, Schnettler F J (1970). J Am Ceram Soc.

[11] Shlyakhtin O A, Tretyakov Y D (1999). J Mater Chem.

[12] Bardakhanov S, Lysenko V, Nomoev A, Trufanov D, Sikalidis C (2011). Ceramic Preparation of Nanopowders and Experimental Investigation of Its Properties. Advances in ceramics – synthesis and characterization, processing and specific applications.

[13] Trusova E A, Trutnev N S, Mortikov E S, Kogan V M, Generalov B M (2009). Catal Ind.

[14] Trusova E A, Trutnev N S, Khrushcheva A A (2015). Method for obtaining nanopowders of crystalline metal oxides using cryotreatment of water-organic sols. Russian Federation Patent.

[15] Generalov M B, Trutnev N S, Onopko K D, Bredikhin N N, Zakrevskij V A, Romanova I A, Platov I V, Trutneva O M (2010). Device for cryogenic granulation of solutions and suspensions. Russian Federation Patent.

[16] Biesingera M C, Payne B P, Grosvenor A P, Lau L W M, Gerson A R, Smart R S C (2011). Appl Surf Sci.

